# Beyond Traditional Body Composition Metrics: Load-Capacity Indices Emerge as Predictors of Cardiometabolic Outcomes—A Systematic Review and Meta-Analysis

**DOI:** 10.1016/j.advnut.2024.100364

**Published:** 2025-01-03

**Authors:** Zhongyang Guan, Marianna Minnetti, Steven B Heymsfield, Eleonora Poggiogalle, Carla M Prado, Marc Sim, Blossom CM Stephan, Jonathan CK Wells, Lorenzo M Donini, Mario Siervo

**Affiliations:** 1 School of Population Health, Faculty of Health Science, Curtin University, Perth, WA, Australia; 2 Department of Experimental Medicine, Sapienza University, Rome, Italy; 3 Pennington Biomedical Research Center, Baton Rouge, LA, United States; 4 Department of Agricultural, Food & Nutritional Science, University of Alberta, Edmonton, AB, Canada; 5 Nutrition and Health Innovation Research Institute, School of Health and Medical Science, Edith Cowan University, Perth, WA, Australia; 6 Medical School, The University of Western Australia, Perth, WA, Australia; 7 Dementia Centre of Excellence, Curtin enAble Institute, Faculty of Health Sciences, Curtin University, Perth, WA, Australia; 8 Childhood Nutrition Research Centre, University College London, London, United Kingdom

**Keywords:** body composition, sarcopenic obesity, cardiometabolic diseases, systematic review, meta-analysis

## Abstract

The adaptive and independent interrelationships between different body composition components have been identified as crucial determinants of disease risk. On the basis of this concept, the load-capacity model of body composition, which utilizes measurements obtained through nonanthropometric techniques such as dual-energy X-ray absorptiometry, was proposed. This model is typically operationalized as the ratio of metabolic load (adipose mass) to metabolic capacity (lean mass). In recent years, a series of load-capacity indices (LCIs) have been utilized to identify abnormal body composition phenotypes such as sarcopenic obesity (SO) and to predict the risk of metabolic, cardiovascular, and cognitive disorders. In this review, we comprehensively review the characteristics of different LCIs used in previous studies, with a specific focus on their applications, especially in identifying SO and predicting cardiometabolic outcomes. A systematic literature search was performed using PubMed, MEDLINE, PsycINFO, Embase, and the Cochrane Library. Two meta-analyses were conducted to *1*) estimate the overall prevalence of SO mapped by LCIs, and *2*) assess the association of LCIs with cardiometabolic outcomes. A total of 48 studies (all observational) were included, comprising 22 different LCIs. Ten studies were included in the meta-analysis of SO prevalence, yielding a pooled prevalence of 14.5% [95% confidence interval (CI): 9.4%, 21.6%]. Seventeen studies were included in the meta-analysis of the association between LCIs and adverse cardiometabolic outcomes, which showed a significant association between higher LCI values and increased risk (odds ratio = 2.22; 95% CI: 1.81, 2.72) of cardiometabolic diseases (e.g. diabetes and metabolic syndrome). These findings suggest that the load-capacity model of body composition could be particularly useful in the identification of SO cases and prediction of cardiometabolic risk. Future longitudinal studies are needed to validate the association of LCIs with chronic cardiometabolic and neurodegenerative diseases.

This systematic review and meta-analysis has been registered with PROSPERO (CRD42024457750).

**Statement of Significance**

The load-capacity model of body composition, which utilizes measurements obtained through non-anthropometric techniques, offers novel insights into understanding the complex interrelationships between different body composition components and their associations with health outcomes. As the first systematic review to synthesize the applications of the load-capacity model of body composition in human research, our study demonstrates the model's potential efficacy for the identification of sarcopenic obesity and the prediction of cardiometabolic risk.

## Introduction

Body composition assessment primarily quantifies the relative amounts of fat mass (FM), lean mass (LM), bone mass, and water content in an individual [1,2]. This information is crucial for evaluating and monitoring nutritional status, predicting disease risk, and guiding therapeutic protocols [[2], [3], [4]]. Body composition measurements are particularly valuable for guiding personalized interventions and tracking changes over time in conditions (e.g. obesity, sarcopenia) or interventions that are likely to impact body composition (e.g. weight loss treatments, dialysis sessions, or chemotherapy) [2,[5], [6], [7]]. Methods for the assessment of body composition differ in terms of theoretical principles, complexity of protocols and analyses, costs, accessibility, and accuracy [2,8]. More accurate, direct methods include MRI, computed tomography (CT), dual-energy X-ray absorptiometry (DXA), deuterium dilution, and air displacement plethysmography, which can be combined in multicompartment models to provide more reliable assessments [4]. Anthropometric indices (e.g. BMI, waist circumference, and waist-to-height ratio) and bioelectrical impedance analysis (BIA) exhibit lower accuracy but are more frequently applied. This is because of their greater accessibility, lower costs, and rapidity of measurements, making them the preferred options in clinical settings and epidemiological studies [2,9,10].

The dynamic and independent interrelationships between FM and LM have been identified as specific determinants of disease risk [11,12]. These relationships underlie the scientific rationale for the development of the load-capacity model of body composition [13]. The load-capacity model is a diagnostic framework that conceptualizes the human body as a balance between metabolically demanding tissues (the “load”) and tissues that support homeostatic metabolic function (the “capacity”) [14]. In this model, adipose tissue, particularly visceral adiposity, is considered the primary “load” because it requires energy for maintenance and can produce inflammatory factors. The “capacity” components include skeletal muscle, liver, and other organs that play crucial roles in glucose regulation, lipid metabolism, and overall metabolic health. This model suggests that health risks may arise when the load exceeds the physiological capacity to compensate effectively, and this burden may be further exacerbated by behavioral factors (i.e. sedentary lifestyle and unbalanced dietary patterns) [14,15]. By analyzing the ratio and distribution of these tissue types, the load-capacity model may provide insights into the regulation of metabolic functions and potential risk for cardiometabolic diseases, including type 2 diabetes, coronary artery disease, and stroke [14,15].

**FIGURE 1:**
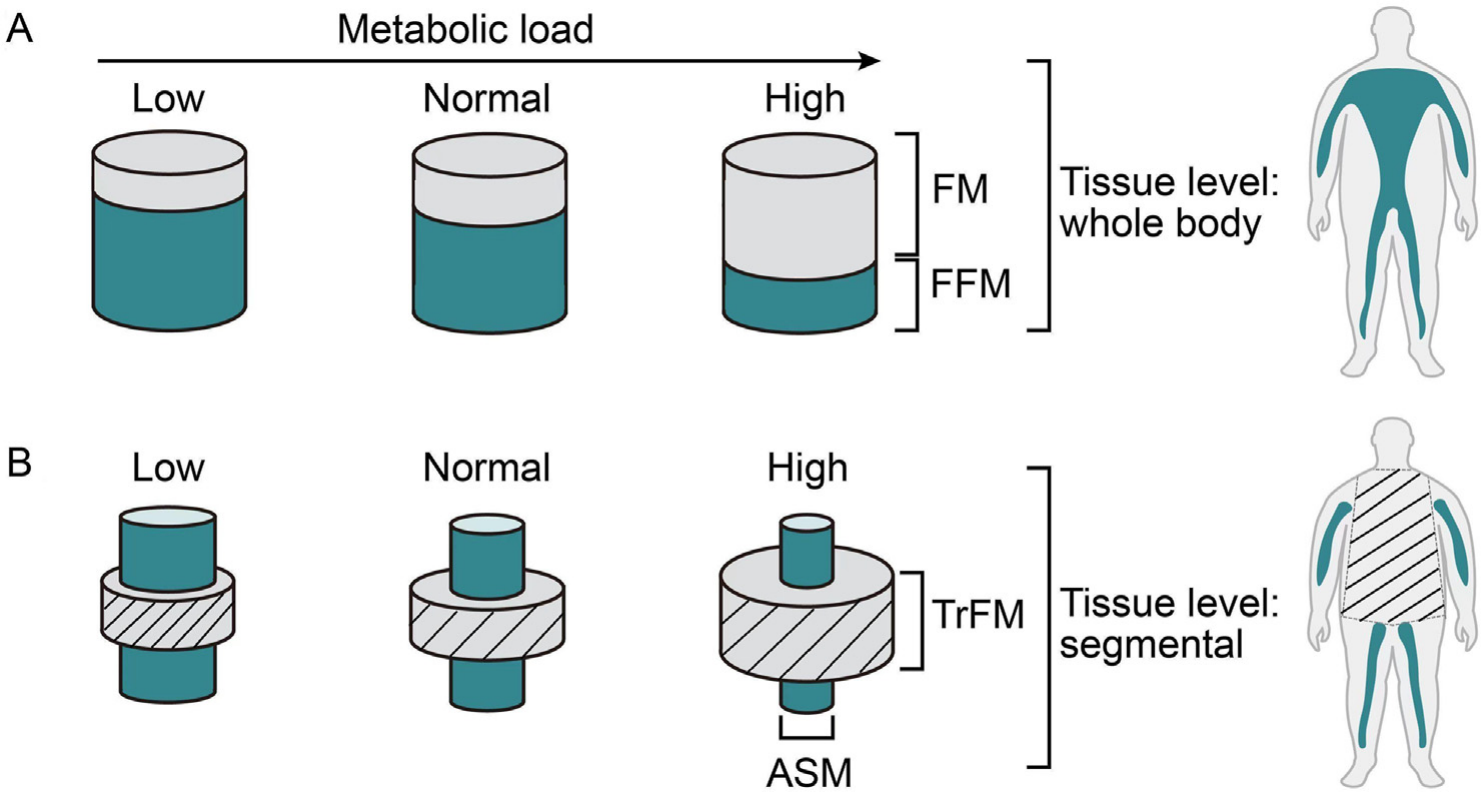
Schematic diagram of the load-capacity model of body composition, based on 2 load-capacity indices (LCIs): (A) whole-body and (B) segmental. The whole-body LCI is based on the ratio of fat mass to fat-free mass (FM/FFM), whereas the segmental LCI is based on the ratio of truncal fat mass to appendicular skeletal muscle mass (TrFM/ASM). The metabolic load has been divided into low, normal, and high categories according to the relative contributions of the 2 components. This diagram was adapted with permission from Siervo et al. [12].

In recent years, there has been growing interest in applying this model to body composition research through the development of a series of load-capacity indices (LCIs), typically based on the ratio of adipose mass to LM [12,[16], [17], [18], [19]]. For instance, Siervo et al. [12] proposed 2 LCIs [whole body: FM/fat-free mass (FFM) ratio and segmental: ratio of truncal fat mass to appendicular skeletal muscle mass (TrFM/ASM)] to identify sarcopenic obesity (SO) cases by assigning metabolic load and metabolic capacity to adipose mass and LM, respectively (Figure 1) [12]. Similarly, other researchers have developed a series of indices, including muscle mass (MM) to FM ratio and FM to LM ratio [19,20]. Although not all these studies explicitly employed the load-capacity model to elucidate these ratios, we propose to categorize all these ratios as LCIs due to their conceptual similarities. To date, no study has systematically reviewed the applications of these LCIs. Therefore, this study aims to comprehensively synthesize the characteristics of different LCIs (i.e. ratio of adipose mass and LM) that have been proposed and evaluate the differences in their *1*) numerators and denominators, *2*) body composition assessment methods, *3*) capacity for identifying SO, and *4*) associations with cardiometabolic health.

## Methods

### Search strategy and study selection

**FIGURE 2:**
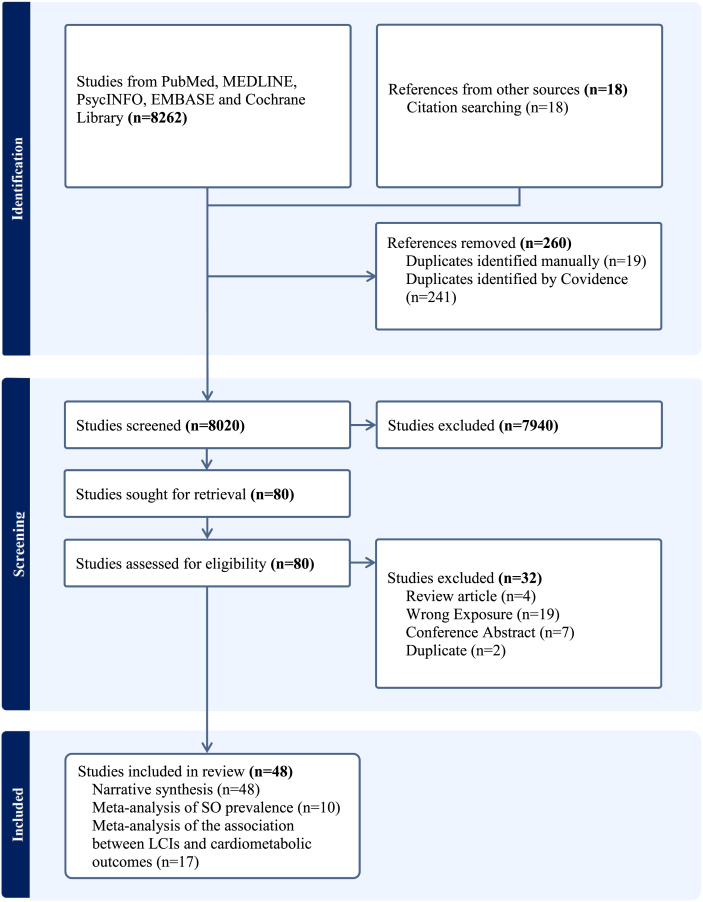
Flowchart of the study selection.

This systematic review is reported in accordance with the PRISMA 2020 guidelines and has been registered on PROSPERO (CRD42024457750) [21]. PubMed, MEDLINE, PsycINFO, Embase, and the Cochrane Library were searched from their inception to 27 March, 2024. The search strategy employed a combination of synonyms and relevant Medical Subject Headings terms for the load-capacity model, ratio, body composition, and SO (Supplemental Table 1). The complete study search and selection process is presented in Figure 2. Titles and abstracts were independently screened in duplicate by 2 reviewers (ZG and MM). Full texts were also independently screened in duplicate by 2 reviewers (ZG and MM). Any disagreements were resolved through discussion with a third reviewer (MS).

We included both observational and experimental studies that met the following criteria: *1*) human participants of any age and health status; *2*) body composition data assessed via nonanthropometric techniques (e.g. BIA, DXA, and CT), from which a ratio measure of different body composition components was derived. Additional elements—namely, *3*) prevalence of SO mapped by LCIs; *4*) investigation of the association between LCIs and health outcomes; or *5*) comparisons among groups categorized by tertile, quartile, quintile, or other predefined cutoffs of LCIs to evaluate the LCI as a health outcome predictor—were not required for inclusion but were noted when present. Studies were excluded if they: *1*) utilized self-reported measures of body composition (e.g. SARC-F); or *2*) review articles or conference abstracts. No restrictions were applied regarding study year or language. In cases where a study was reported in multiple publications, we selected the publication that provided outcomes and information most relevant to our research question, or the one published in a more appropriate format (e.g. an original research article rather than a research letter).

### Data extraction and quality appraisal

We collected the following information from each included study: the first author’s surname, year of publication, country, study design, sample size, population characteristics (e.g. age, sex, race/ethnicity, and BMI), body composition assessment methods, definition of the load-capacity model, primary outcome (SO prevalence), and secondary outcome (association of LCIs with health outcomes), as presented in Supplemental Table 2 and Supplemental Table 3. The Risk Of Bias In Non-randomized Studies of Exposures (ROBINS-E) tool was used to evaluate the risk of bias in the included studies [22,23]. Two reviewers (ZG and MM) independently conducted the data extraction and quality appraisal, with any discrepancies being resolved through discussion with a third reviewer (MS).

### Data synthesis and statistical analysis

**FIGURE 3:**
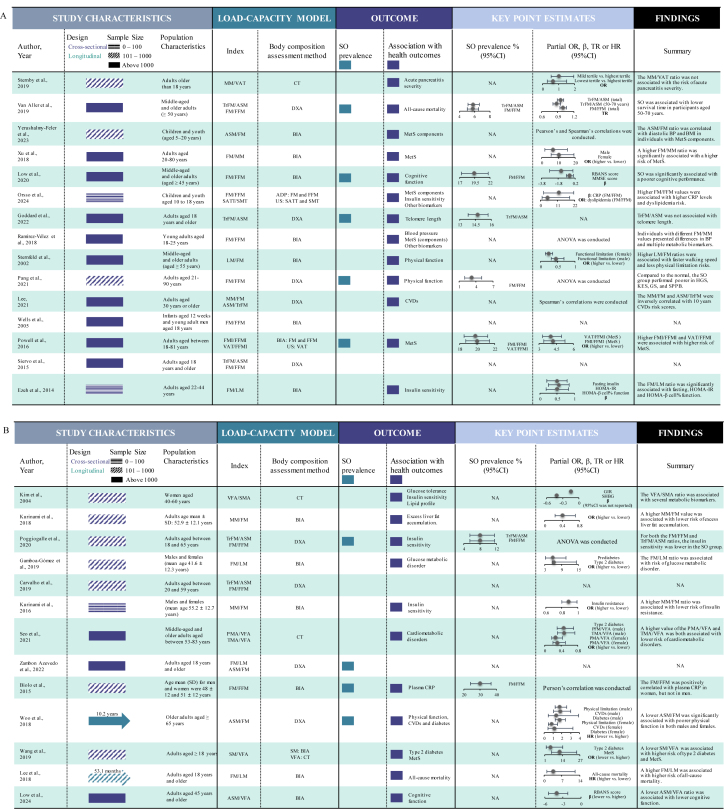

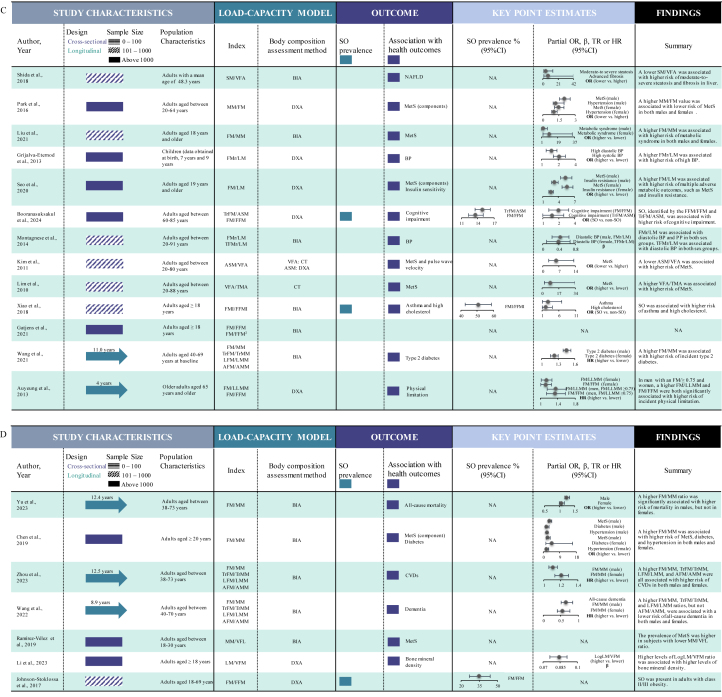
Graphical Overview for Evidence Reviews (GOfER) diagram. ADP, air displacement plethysmography; AFM, arm fat mass; AMM, arm muscles mass; ANOVA, analysis of variance; ASM, appendicular skeletal muscle mass; BIA, bioelectrical impedance analysis; BP, blood pressure; CI, confidence interval; CT, computed tomography; CVD, cardiovascular disease, DXA, dual energy X-ray absorptiometry; FFM, fat-free mass; FFMI, fat-free mass index; FM, fat mass; FMI, fat mass index; FMr/LM, fat mass standardized residuals modeled on lean mass; GIR, glucose-area under the curve/insulin-area under the curve; HR, hazard ratio; LFM, leg fat mass; LLMM, lower-limb muscle mass; LM, lean mass; MetS, metabolic syndrome; MM, muscle mass; NA, not applicable; NAFLD, nonalcoholic fatty liver disease; OR, odds ratio; PMA, paravertebral muscle area; PP, pulse pressure; RBANS, Repeatable Battery for the Assessment of Neuropsychological Status; SATT, subcutaneous adipose tissue thickness; SHBG, sex hormone-binding globulin; SM, skeletal muscle mass; SMA, skeletal muscle area; SO, sarcopenic obesity; TFMr/LM, trunk fat mass residual for lean mass; TMA, thigh muscle area; TR, time ratio; TrFM, truncal fat mass; TrMM, truncal muscle mass; US, ultrasound; VAT, visceral adipose tissue; VFA, visceral fat area; VFL, visceral fat level; VFM, visceral fat mass.

Key characteristics of the included studies were summarized in a Graphical Overview for Evidence Reviews diagram (Figure 3), and overall findings were narratively synthesized. Two distinct meta-analyses were also conducted. First, we pooled the point estimates of studies that reported SO prevalence using LCIs. Second, we pooled the point estimates of the association between LCIs and cardiometabolic outcomes. All LCIs were standardized to represent the ratio of metabolic load to metabolic capacity. Reciprocal ratios, such as FM/FFM and FFM/FM, were considered the same LCI. Similarly, ratios with different numerators and denominators but mathematically equivalent values were also considered the same LCI [e.g. FM/FFM and fat mass index (FM in kg/m^2^)/FFMI (FFM in kg/m^2^)]. The odds ratio (OR), accompanied by 95% confidence interval (CI), was used as the common measure of association between LCIs and disease risk across the included studies. We pooled the adjusted ORs comparing higher LCIs with lower LCIs (reference category). Hazard ratios (HRs) from longitudinal studies were considered approximately equivalent to ORs when calculating the pooled estimates [24]. In both meta-analyses, for studies that exclusively reported estimates for subgroups (i.e. males and females), we calculated the overall population estimates by aggregating subgroup estimates using the fixed-effects model. For studies that categorized the LCIs into >2 groups (e.g. quartiles), we derived the overall point estimates by synthesizing subgroup estimates using the random-effects model. Inter-study heterogeneity was evaluated using the *I*^2^ statistic [25]. The random-effects model was adopted as the pooling method if *I*^2^ ≥ 50%; otherwise, the fixed-effects model was used. Subgroup analyses stratified by LCIs and cardiometabolic outcome categories were conducted to mitigate inter-study heterogeneity. Publication bias for both meta-analyses was assessed *1*) visually, by examining funnel plots for signs of asymmetry, and *2*) statistically, using Egger’s regression tests and the Begg and Mazumdar rank correlation test. If publication bias was present, the trim-and-fill and the precision-effect test and precision-effect estimate with standard error (PET-PEESE) methods were used to adjust the pooled estimates [26,27]. Furthermore, we conducted sensitivity analysis by re-running the meta-analyses with robust variance estimation (RVE) to account for dependencies of point estimates included in a single model, because several studies included in both meta-analyses involved multiple point estimates [28,29]. An additional sensitivity analysis was performed with the “leave-one-out” method, by systematically removing each point estimate and re-calculating the pooled estimates. All statistical analyses were performed using Stata, version 18.0 (StataCorp LLC) and R, version 4.4.1. *P* values <0.05 were considered statistically significant.

## Results

### Narrative synthesis

The flowchart of study search and selection is presented in Figure 2. Our systematic search yielded 8262 articles from databases, supplemented by 18 articles from reference lists of the included studies. Subsequently, 48 studies involving 20 countries were included in this review [12,[16], [17], [18], [19], [20],[30], [31], [32], [33], [34], [35], [36], [37], [38], [39], [40], [41], [42], [43], [44], [45], [46], [47], [48], [49], [50], [51], [52], [53], [54], [55], [56], [57], [58], [59], [60], [61], [62], [63], [64], [65], [66], [67], [68], [69], [70], [71]]. All 48 included studies were observational in design, comprising 41 cross-sectional and 7 longitudinal studies. Twenty-two LCIs were employed across the included studies (Figure 3). The most frequently utilized LCIs were FM/FFM (16 studies), FM/MM (11 studies), TrFM/ASM (7 studies), and FM/LM (6 studies). The most frequently applied nonanthropometric body composition assessment techniques were BIA (24 studies), DXA (17 studies), and CT (6 studies).

Twelve studies estimated the prevalence of SO, of which 10 used LCIs for identification and were included in the meta-analysis for prevalence [18,32,[35], [36], [37],^41^,^47^,^57^,^61^,^71^]. The SO prevalence mapped by LCIs ranged from 3.2% to 50.7%. Forty studies explored the association between LCIs and health outcomes, including adverse cardiometabolic outcomes (*n* = 26), poor physical function (*n* = 4), poor cognitive function (*n* = 4), and mortality (*n* = 3). Seventeen studies that reported the point effect sizes of interest (ORs and HRs) were included in the meta-analysis examining the association between LCIs and adverse cardiometabolic outcomes, including diabetes, metabolic syndrome (MetS), dyslipidemia, and insulin resistance [16,18,34,42,44,45,48,49,53,54,56,[59], [60], [61],^63^,^66^,^67^].

### Quality of studies

The risk of bias, assessed using ROBINS-E, was categorized as low, moderate (some concerns), or high. Of the 48 observational studies, 25 were assessed as having a low risk of bias, and 17 were assessed as having a moderate risk of bias (Supplemental Figure 1). The remaining 6 studies were assessed as having a high risk of bias, primarily due to inadequate adjustment for potential confounders and insufficient information regarding postexposure interventions. None of the studies included in the meta-analyses was assessed as having a high risk of bias.

### Meta-analyses

**FIGURE 4:**
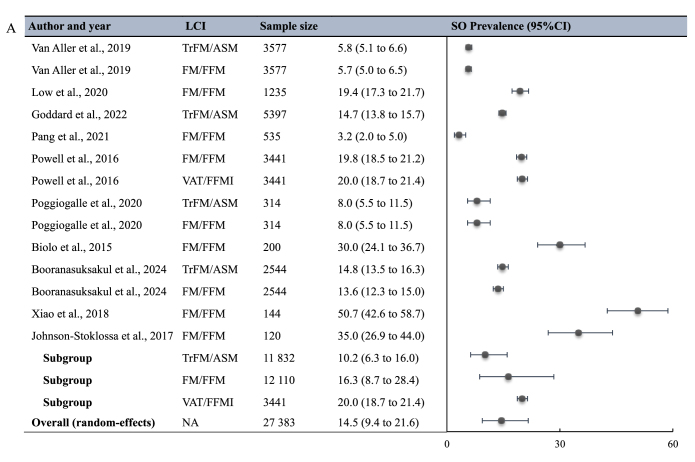

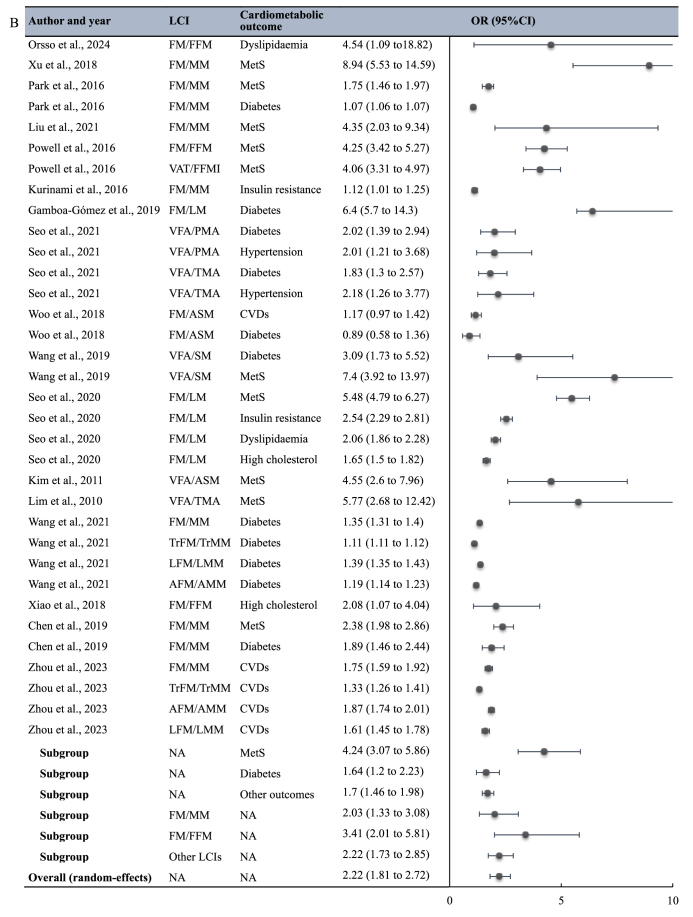
Meta-analysis of the (A) SO prevalence (%) and (B) the association of LCIs with cardiometabolic outcomes. Heterogeneity test result for meta-analysis (A): *I*^2^ = 99.47%, *P* < 0.001. Heterogeneity test result for meta-analysis (B): *I*^2^ = 99.95%, *P* < 0.001. LCI, load-capacity index; SO, sarcopenic obesity.

Ten studies comprising 14 point estimates and involving 27,383 participants (aged ≥18 y) were included in the meta-analysis for LCI-assessed SO prevalence (Figure 4A). Of those, 4 employed TrFM/ASM [32,36,41,57], 9 employed FM/FFM [18,32,35,37,41,47,57,61,71], and 1 employed visceral adipose tissue (VAT)/FFMI [18]. The pooled SO prevalence was 14.5% (95% CI: 9.4%, 21.6%; *I*^2^ = 99.47%). The pooled prevalences differed significantly among the TrFM/ASM group (10.2%; 95% CI: 6.3%, 16.0%), FM/FFM group (16.3%; 95% CI: 8.7%, 28.4%), and VAT/FFMI group (20.0%; 95% CI: 18.7%, 21.4%), with *P* < 0.001 across subgroups.

Seventeen studies comprising 40 point estimates were included in the meta-analysis examining the association of LCIs with adverse cardiometabolic outcomes (Figure 4B), with a pooled OR (higher LCI compared with lower LCI) of 2.22 (95% CI: 1.81, 2.72; *I*^2^ = 99.95%). Nine studies specifically examined the association between LCIs and MetS risk [18,34,49,53,54,56,59,60,66], demonstrating a stronger association (OR = 4.24; 95% CI: 3.07, 5.86). In contrast, 7 studies explored the association between LCIs and diabetes risk [42,45,48,49,53,63,66], showing a weaker association (OR = 1.64; 95% CI: 1.20, 2.23). Seven studies reported the association of LCIs with risk of other cardiometabolic outcomes, with a pooled OR = 1.70 (95% CI: 1.46, 1.98) [16,44,45,48,56,61,67]. The difference across subgroups was significant (*P* < 0.001). FM/MM and FM/FFM were the most frequently utilized LCIs in the included studies. Three studies utilized FM/FFM to examine the association between LCIs and cardiometabolic health, demonstrating a stronger association (OR = 3.41; 95% CI: 2.01, 5.81) [16,18,61]. In contrast, 7 studies utilized FM/MM, showing a weaker association (OR = 2.03; 95% CI: 1.33, 3.08) [34,44,53,54,63,66,67]; however, the difference across subgroups was not significant (*P* = 0.53).

For the meta-analysis of SO prevalence, no significant publication bias was identified (Supplemental Figure 2). Conversely, publication bias for the meta-analysis of the association between LCIs and adverse cardiometabolic outcomes was detected, according to the results of Egger’s regression tests and the Begg and Mazumdar rank correlation test (Supplemental Figure 2). The trim-and-fill method to symmetrize the funnel plot did not change the pooled effect size; however, when the PET-PEESE method was applied, the effect size adjusted by the small study effects was slightly lower (OR = 1.92; 95% CI: 1.56, 2.37), underscoring a bias toward publishing studies with stronger associations. Sensitivity analyses indicated that the pooled estimate for SO prevalence (16.3%; 95% CI: 9.5%, 26.5%) and OR (2.56; 95% CI: 1.79, 3.65) were higher after applying RVE (Supplemental Figure 3). However, the results for both meta-analyses remained consistent and did not exhibit significant alteration upon the exclusion of any point estimates (Supplemental Table 4).

## Discussion

### Main findings

This study represents the first systematic review and meta-analysis to comprehensively investigate the applications of the load-capacity model of body composition, focusing specifically on its definition, the prevalence of SO identified by the LCIs, and the association between LCIs and cardiometabolic outcomes. We identified 22 LCIs, with FM/FFM, TrFM/ASM, FM/MM, and FM/LM being the most frequently employed. Our meta-analysis results indicated that the overall prevalence of SO identified by LCIs was 14.5% (95% CI: 9.4%, 21.6%). Meta-analysis also demonstrated that higher LCI values were associated with a 122% increase (95% CI: 81%, 172%) in the odds of experiencing adverse cardiometabolic outcomes. Specifically, higher LCI values were associated with increased odds of diabetes (64%; 95% CI: 20%, 123%) and MetS (324%; 95% CI: 207%, 486%).

### Prevalence of SO

In the 10 studies that reported the LCI-mapped SO prevalence estimates, SO was typically identified by LCIs values exceeding specific cut-off points, which were derived using 2 approaches. One approach employed a cut-off value of 0.8 for the FM/FFM ratio [35,37,47], whereas the second utilized the 85th percentiles of the age-, sex-, and BMI-specific LCIs distributions (FM/FFM and TrFM/ASM) derived from the NHANES 1999–2004 DXA data [12]. Over recent decades, the utilization of numerous definitions and diagnostic criteria for SO has significantly contributed to divergent prevalence estimates. Several meta-analyses have reported global SO prevalences among older adults, with pooled prevalences ranging from 9% to 14% [[72], [73], [74]]. Our meta-analysis yielded pooled estimates for TrFM/ASM subgroup prevalence (10.2%) that are consistent with this range.

In 2022, the Sarcopenic Obesity Global Leadership Initiative (SOGLI), launched by the European Society for Clinical Nutrition and Metabolism and the European Association for the Study of Obesity (ESPEN-EASO), achieved consensus on the definition and diagnostic algorithm for SO, marking a significant step toward standardization in the field [75,76]. The SOGLI also proposed exploring the validity of the load-capacity model of body composition in SO identification and its association with health outcomes [77]. However, a comprehensive comparison between the SO prevalences mapped by the LCIs and ESPEN-EASO criteria was not conducted in this review, because no meta-analysis has yet reported the pooled prevalence of SO identified using the ESPEN-EASO diagnostic criteria, to the best of our knowledge. A recent study utilizing NHANES data reported an SO prevalence of 15.0% among middle-aged and older adults (≥50 y) based on the ESPEN-EASO criteria [78]. This prevalence is comparable with our pooled estimates for the overall prevalence and FM/FFM subgroup prevalence, and NHANES data were also used in several studies included in our meta-analyses. Additionally, several population-based studies reported prevalences mapped by the ESPEN-EASO criteria that were close to our pooled prevalence for the TrFM/ASM subgroup [79,80]. Consequently, the FM/FFM and TrFM/ASM ratios might be promising for the identification of SO. Unlike the load-capacity model of body composition utilized to identify SO cases, the ESPEN-EASO criteria incorporate both alterations in body composition (reduced MM and increased %FM) and skeletal muscle function. Therefore, these 2 LCIs could be integrated within the ESPEN-EASO criteria, and they may have broader applicability in scenarios where assessment of muscle function is not feasible. Nonetheless, further validation of the VAT/FFMI ratio is needed, because only 1 study in our analysis utilized this LCI to identify SO.

### LCIs and cardiometabolic health

In the subgroup analysis examining the association between LCIs and cardiometabolic health, the association of LCIs with MetS was significantly stronger than with other cardiometabolic outcomes, including insulin resistance and hypertension. In contrast, we did not observe any significant differences in the associations of different LCIs with cardiometabolic health, although the FM/FFM subgroup showed a higher pooled estimate, and the FM/MM subgroup showed a lower pooled estimate. Consequently, we were unable to conclusively determine which specific LCI serves as the superior predictor of cardiometabolic risk.

Several interrelated mechanisms may explain the association between higher levels of LCIs and increased cardiometabolic risk. Among these, insulin resistance—a well-established risk factor for cardiovascular diseases (CVDs) and main MetS components (e.g. hypertension, hyperglycemia, dyslipidemia, and abdominal obesity)—may serve as a cornerstone [[81], [82], [83]]. Given that insulin-induced glucose uptake primarily occurs in skeletal muscle, decreased MM may reduce insulin sensitivity [84,85]. Increased adipose tissue mass, particularly abdominal adipose tissue, is also closely related to lower insulin sensitivity [[85], [86], [87]]. However, lower FFM may also index smaller organ size and reduced capacity for homeostasis (e.g. glucose regulation by muscles), which may be an alternative mechanism contributing to elevated cardiometabolic risk [13]. Therefore, in the context of lower LCI values, both a relative decrease in LM and a relative increase in adipose mass can be associated with insulin resistance. On the other hand, lower LCI values may be associated with reduced physical activity and decreased resting metabolic rate, consequently exacerbating adipose tissue accumulation and muscle loss [88,89]. Unbalanced dietary patterns (e.g. high-calorie intake and low-protein consumption) and anabolic resistance may also contribute to this association by leading to increased adipose mass or decreased LM [[90], [91], [92]]. Additionally, higher LCI levels and cardiometabolic diseases may share other pathophysiological mechanisms, including chronic inflammation, oxidative stress, and hormonal changes [93].

### Whole body LCIs and segmental LCIs

A critical finding from our meta-analysis on SO prevalences mapped by LCIs was the significant differences in SO prevalences identified between a commonly used whole-body LCI (FM/FFM) and a segmental LCI (TrFM/ASM) (Figure 1). These 2 methods possess distinct advantages and limitations. Implementation of the whole-body LCI (FM/FFM) is generally more straightforward in clinical settings, requiring less complex measurements and demonstrating greater feasibility with basic body composition assessment tools such as BIA. However, it may not capture regional fat distribution patterns that are particularly relevant to cardiometabolic risk [12]. On the other hand, the segmental LCI (TrFM/ASM) was developed based on the well-established association of abdominal fat accumulation (TrFM) with metabolic impairment and ASM with oxidative functions and metabolic flexibility [94]. This physiological rationale suggests that the segmental approach may offer more precise insights into metabolic health. However, it normally requires more sophisticated measurement techniques such as DXA and may be less accessible in routine clinical practice. Therefore, when utilizing these indices, accessibility of technology and cost-effectiveness should be particularly considered.

### Strengths and limitations

The present review has several strengths. First, the exhaustive search of 5 databases, without restrictions on population, study design, or language, enabled a comprehensive synthesis of results across diverse sociodemographic and methodological contexts. Second, we utilized the LCIs to categorize all ratios of adipose mass and LM. This unified format provides a standardized reference and facilitates future research. For instance, future meta-analyses could refer to our methodology when synthesizing results related to these ratios. Third, the subgroup analyses provided evidence regarding the efficacy of 2 specific LCIs (FM/FFM and TrFM/ASM) in identifying SO cases, as well as the predictive capacity of LCIs for specific cardiometabolic diseases, including MetS and diabetes. Last, we conducted meta-analyses with RVE as sensitivity analyses, offering an opportunity to evaluate the robustness of our findings. The RVE allowed us to account for the dependency of point estimates, potentially yielding unbiased pooled estimates. However, it is important to note that both meta-analyses included a relatively small number of studies, which might limit the accuracy of the RVE [28]. Therefore, we interpreted the RVE results cautiously, considering them complementary to our primary analyses using conventional meta-analytic techniques rather than as definitive.

Several limitations warrant consideration in the interpretation of our findings. First, all the included studies were observational in nature, with a large number also being cross-sectional. This cross-sectional design precludes the assessment of temporal changes in LCIs and their relationship with the progression of related cardiometabolic outcomes. Therefore, although we observed significant associations between LCIs and cardiometabolic risk, these findings may primarily reflect LCIs’ role as risk indicators rather than as definitive markers of advanced cardiometabolic states. Second, only 1 study included in our meta-analyses was conducted in a low- and middle-income country (LMIC) [34], thus precluding subgroup meta-analyses based on this metric. Similarly, the impact of age, sex, race/ethnicity, and other demographic factors that have been reported to influence the prevalence of SO and the risk of the investigated cardiometabolic outcomes could not be stratified across both meta-analyses [74,95,96]. These factors could influence our pooled estimates of SO prevalence and the associations between LCIs and cardiometabolic outcomes, thereby affecting the generalizability of our findings across different populations. For instance, older adults demonstrated increased susceptibility to SO and cardiometabolic outcomes (e.g. CVDs) [74,97]. Notably, the pooled prevalence of SO identified by the LCIs was comparable with that reported in previous meta-analyses [[72], [73], [74]]. However, the previously reported prevalences pertained specifically to older adults, whereas our pooled prevalence involved populations with a wider age range (i.e. ≥18 y). Third, we were unable to conduct subgroup meta-analyses stratified according to different body composition assessment techniques employed in the included studies. As previously discussed, different body composition techniques (e.g. BIA, DXA, and CT) differ in measurement accuracy and precision, which could potentially introduce systematic variations in the derived LCIs [2,9,10]. Future meta-analyses with sufficient studies using different assessment methods should consider conducting method-specific analyses to validate and extend our findings. Fourth, substantial heterogeneity persisted in both meta-analyses after subgroup analyses. This heterogeneity may be attributed to the diversity of sociodemographic characteristics of participants, body composition assessment methods, LCI cut-off values, and definitions and diagnostic criteria for the investigated cardiometabolic outcomes.

### Implications for future research

Although this meta-analysis provides an extensive overview of the existing evidence on the SO prevalence mapped by LCIs and the association of LCIs with cardiometabolic outcomes, the limitations present in the included studies necessitate cautious interpretation of our findings and underscore the need for more high-quality studies. Future research should focus on longitudinal studies to explore the causal relationship of LCIs with cardiometabolic outcomes, especially MetS and diabetes, and other health outcomes (e.g. neurodegenerative diseases), using widely accepted criteria to identify these conditions. More population-based studies, especially those focusing on populations with specific sociodemographic characteristics (e.g. older adults and LMICs residents), are also warranted to evaluate the superiority of specific LCIs for the identification of SO cases and prediction of cardiometabolic risk. Furthermore, future studies could also investigate the dose-response relationship between LCIs and adverse cardiometabolic outcomes, which may provide novel insights into the role of the dynamic and independent interrelationships between adipose mass and LM in the onset and progression of cardiometabolic diseases.

## Conclusions

As the first review synthesizing the applications of the load-capacity model of body composition in human research, our study highlights the model’s capability for the identification of SO and the prediction of cardiometabolic risk. Our findings and the methodology for categorizing the ratios of adipose mass and LM may serve as a stepping-stone for future research to validate the association of LCIs with cardiometabolic diseases and to evaluate the efficacy of specific LCIs for SO identification and cardiometabolic disease prediction.

## Author contributions

The authors’ responsibilities were as follows – M Siervo, LMD: designed the research; ZG, MM, M Siervo: screened the literature; ZG, MM: conducted the data extraction; ZG, MM, M Siervo: performed the risk of bias assessment; ZG: conducted the statistical analysis; ZG, M Siervo: drafted the manuscript; all authors: contributed to the protocol and the critical review of the manuscript; and all authors: read and approved the final manuscript.

## Funding

ZG is supported by the Curtin Higher Degree by Research (HDR) Scholarship and the Dementia Centre of Excellence (DCE) and Curtin enAble Institute Seed Funding. LMD, MM, and EP acknowledge the support of grant PE00000003 (decree 1550, 11.10.2022) (“ON Foods – Research and innovation network on food and nutrition Sustainability, Safety and Security – Working ON Foods”) from the Italian Ministry of University and Research (Sapienza University CUP B53C22004030001) under the National Recovery and Resilience Plan (NRRP), funded by the European Union – NextGenerationEU. CMP is partially funded through the Canada Research Chairs Program. The salary of M Sim is supported by an Emerging Leader Fellowship from the Future Health Research and Innovation Fund, Department of Health, Western Australia.

## Data availability

All data analyzed or generated during this study are included in this published article and the accompanying supplementary data files.

## Conflict of interest

CMP reports honoraria and/or paid consultancy from Abbott Nutrition, Nutricia, Nestlé Health Science, Pfizer, and AMRA medical; and investigator-initiated funding from Almased. The other authors declare no conflicts of interest.

## References

[1] Lee S.Y., Gallagher D. (2008). Assessment methods in human body composition. Curr. Opin. Clin. Nutr. Metab. Care..

[2] Holmes C.J., Racette S.B. (2021). The utility of body composition assessment in nutrition and clinical practice: an overview of current methodology. Nutrients.

[3] Amato M.C., Guarnotta V., Giordano C. (2013). Body composition assessment for the definition of cardiometabolic risk. J. Endocrinol. Invest..

[4] Sullivan K., Hornikel B., Holmes C.J., Esco M.R., Fedewa M.V. (2022). Validity of a 3-compartment body composition model using body volume derived from a novel 2-dimensional image analysis program. Eur. J. Clin. Nutr..

[5] Jeanmaire C., Mazières B., Verrouil E., Bernard L., Guillemin F., Rat A.-C. (2018). Body composition and clinical symptoms in patients with hip or knee osteoarthritis: results from the KHOALA cohort. Semin. Arthritis Rheum..

[6] Awad S., Tan B.H., Cui H., Bhalla A., Fearon K.C.H., Parsons S.L. (2012). Marked changes in body composition following neoadjuvant chemotherapy for oesophagogastric cancer. Clin. Nutr..

[7] Pellicano R., Strauss B.J., Polkinghorne K.R., Kerr P.G. (2011). Longitudinal body composition changes due to dialysis. Clin. J. Am. Soc. Nephrol..

[8] Ceniccola G.D., Castro M.G., Piovacari S.M.F., Horie L.M., Corrêa F.G., Barrere A.P.N. (2019). Current technologies in body composition assessment: advantages and disadvantages. Nutrition.

[9] Andreoli A., Garaci F., Cafarelli F.P., Guglielmi G. (2016). Body composition in clinical practice. Eur. J. Radiol..

[10] Borga M., West J., Bell J.D., Harvey N.C., Romu T., Heymsfield S.B. (2018). Advanced body composition assessment: from body mass index to body composition profiling. J. Investig. Med..

[11] Prado C.M., Siervo M., Mire E., Heymsfield S.B., Stephan B.C., Broyles S. (2014). A population-based approach to define body-composition phenotypes. Am. J. Clin. Nutr..

[12] Siervo M., Prado C.M., Mire E., Broyles S., Wells J.C., Heymsfield S. (2015). Body composition indices of a load–capacity model: gender- and BMI-specific reference curves. Public Health Nutr.

[13] Wells J.C.K. (2018). The capacity–load model of non-communicable disease risk: understanding the effects of child malnutrition, ethnicity and the social determinants of health. Eur. J. Clin. Nutr..

[14] Wells J.C.K. (2009). Historical cohort studies and the early origins of disease hypothesis: making sense of the evidence: Workshop on ‘Nutritional models of the developmental origins of adult health and disease’. Proc. Nutr. Soc..

[15] Wells J.C.K. (2011). The thrifty phenotype: an adaptation in growth or metabolism?. Am. J. Hum. Biol..

[16] Orsso C.E., Vieira F.T., Basuray N., Duke R.L., Pakseresht M., Rubin D.A. (2024). The metabolic load-capacity model and cardiometabolic health in children and youth with obesity. Pediatr. Obes..

[17] Ramírez-Vélez R., Carrillo H.A., Correa-Bautista J.E., Schmidt-RioValle J., González-Jiménez E., Correa-Rodríguez M. (2018). Fat-to-muscle ratio: a new anthropometric indicator as a screening tool for metabolic syndrome in young Colombian people. Nutrients.

[18] Powell M., Lara J., Mocciaro G., Prado C.M., Battezzati A., Leone A. (2016). Association between ratio indexes of body composition phenotypes and metabolic risk in Italian adults. Clin. Obes..

[19] Ezeh U., Pall M., Mathur R., Azziz R. (2014). Association of fat to lean mass ratio with metabolic dysfunction in women with polycystic ovary syndrome. Hum. Reprod..

[20] Kurinami N., Sugiyama S., Morita A., Yoshida A., Hieshima K., Miyamoto F. (2018). Ratio of muscle mass to fat mass assessed by bioelectrical impedance analysis is significantly correlated with liver fat accumulation in patients with type 2 diabetes mellitus. Diabetes Res. Clin. Pract..

[21] Page M.J., McKenzie J.E., Bossuyt P.M., Boutron I., Hoffmann T.C., Mulrow C.D. (2021). The PRISMA 2020 statement: an updated guideline for reporting systematic reviews. BMJ.

[22] Higgins J.P.T., Morgan R.L., Rooney A.A., Taylor K.W., Thayer K.A., Silva R.A. (2024). A tool to assess risk of bias in non-randomized follow-up studies of exposure effects (ROBINS-E). Environ. Int..

[23] McGuinness L.A., Higgins J.P.T. (2021). Risk-of-bias VISualization (robvis): an R package and Shiny web app for visualizing risk-of-bias assessments. Res. Synth. Methods..

[24] Zhang J., Yu K.F. (1998). What’s the relative risk?: a method of correcting the odds ratio in cohort studies of common outcomes. JAMA.

[25] Higgins J.P.T., Thompson S.G. (2002). Quantifying heterogeneity in a meta-analysis. Stat. Med..

[26] Duval S., Tweedie R. (2000). Trim and fill: a simple funnel-plot–based method of testing and adjusting for publication bias in meta-analysis. Biometrics.

[27] Stanley T.D., Doucouliagos H. (2014). Meta-regression approximations to reduce publication selection bias. Res. Synth. Methods..

[28] Hedges L.V., Tipton E., Johnson M.C. (2010). Robust variance estimation in meta-regression with dependent effect size estimates. Res. Synth. Methods..

[29] Moeyaert M., Ugille M., Natasha Beretvas S., Ferron J., Bunuan R., Van Den Noortgate W. (2017). Methods for dealing with multiple outcomes in meta-analysis: a comparison between averaging effect sizes, robust variance estimation and multilevel meta-analysis. Int. J. Soc. Res. Methodol..

[30] Sternfeld B., Ngo L., Satariano W.A., Tager I.B. (2002). Associations of body composition with physical performance and self-reported functional limitation in elderly men and women. Am. J. Epidemiol..

[31] Sternby H., Mahle M., Linder N., Erichson-Kirst L., Verdonk R.C., Dimova A. (2019). Mean muscle attenuation correlates with severe acute pancreatitis unlike visceral adipose tissue and subcutaneous adipose tissue. United European Gastroenterol. J..

[32] Van Aller C., Lara J., Stephan B.C.M., Donini L.M., Heymsfield S., Katzmarzyk P.T. (2019). Sarcopenic obesity and overall mortality: results from the application of novel models of body composition phenotypes to the National Health and Nutrition Examination Survey 1999–2004. Clin. Nutr..

[33] Yerushalmy-Feler A., Kassner O., Frank Y., Moran-Lev H., Anafy A., Levy D. (2023). Body composition in pediatric celiac disease and metabolic syndrome component risk—an observational study. Pediatr. Res..

[34] Xu K., Zhu H.J., Chen S., Chen L., Wang X., Zhang L.Y. (2018). Fat-to-muscle ratio: a new anthropometric indicator for predicting metabolic syndrome in the Han and Bouyei populations from Guizhou Province, China, Biomed. Environ. Sci..

[35] Low S., Goh K.S., Ng T.P., Ang S.F., Moh A., Wang J. (2020). The prevalence of sarcopenic obesity and its association with cognitive performance in type 2 diabetes in Singapore. Clin. Nutr..

[36] Goddard T., Tsintzas K., Stephan B.C.M., Prado C.M., Mazidi M., Siervo M. (2022). Sarcopenic obesity is associated with telomere shortening: findings from the NHANES 1999–2002. Int. J. Obes. (Lond)..

[37] Pang B.W.J., Wee S.L., Lau L.K., Jabbar K.A., Seah W.T., Ng D.H.M. (2021). Obesity measures and definitions of sarcopenic obesity in Singaporean adults – the Yishun Study. J. Frailty Aging..

[38] Lee K. (2021). Bone mediated and moderated the associations between sarcopenic obesity indices and cardiovascular disease risk scores. Calcif. Tissue Int..

[39] Wells J.C.K., Victora C.G. (2005). Indices of whole-body and central adiposity for evaluating the metabolic load of obesity. Int. J. Obes. (Lond)..

[40] Kim C.S., Nam J.Y., Park J.S., Kim D.M., Yoon S.J., Ahn C.W. (2004). The correlation between insulin resistance and the visceral fat to skeletal muscle ratio in middle-aged women. Yonsei Med. J..

[41] Poggiogalle E., Mendes I., Ong B., Prado C.M., Mocciaro G., Mazidi M. (2020). Sarcopenic obesity and insulin resistance: application of novel body composition models. Nutrition.

[42] Gamboa-Gómez C.I., Simental-Mendía L.E., Rodríguez-Morán M., Guerrero-Romero F. (2019). The fat-to-lean mass ratio, a novel anthropometric index, is associated to glucose metabolic disorders. Eur. J. Intern. Med..

[43] Carvalho C.J.D., Longo G.Z., Juvanhol L.L., Kakehasi A.M., Pereira P.F., Segheto K.J. (2019). Body composition indices in Brazilian adults: age-specific and sex-specific percentile curves. Arch. Endocrin. Metab..

[44] Kurinami N., Sugiyama S., Yoshida A., Hieshima K., Miyamoto F., Kajiwara K. (2016). Correlation of body muscle/fat ratio with insulin sensitivity using hyperinsulinemic-euglycemic clamp in treatment-naïve type 2 diabetes mellitus. Diabetes Res. Clin. Pract..

[45] Seo H.S., Lee H., Kim S., Lee S.K., Lee K.Y., Kim N.H. (2021). Paravertebral muscles as indexes of sarcopenia and sarcopenic obesity: comparison with imaging and muscle function indexes and impact on cardiovascular and metabolic disorders. AJR Am. J. Roentgenol..

[46] Zambon Azevedo V., Ponnaiah M., Bel Lassen P., Ratziu V., Oppert J.-M. (2022). A diagnostic proposal for sarcopenic obesity in adults based on body composition phenotypes. Clin. Nutr. ESPEN.

[47] Biolo G., Di Girolamo F.G., Breglia A., Chiuc M., Baglio V., Vinci P. (2015). Inverse relationship between “a body shape index” (ABSI) and fat-free mass in women and men: insights into mechanisms of sarcopenic obesity. Clin. Nutr..

[48] Woo J., Leung J. (2018). Sarcopenic obesity revisited: insights from the Mr and Ms Os cohort. J. Am. Med. Dir. Assoc..

[49] Wang Q., Zheng D., Liu J., Fang L., Li Q. (2019). Skeletal muscle mass to visceral fat area ratio is an important determinant associated with type 2 diabetes and metabolic syndrome. Diabetes Metab. Syndr. Obes..

[50] Lee H.-S., Kim S.G., Kim J.-K., Lee Y.K., Noh J.W., Oh J. (2018). Fat-to-lean mass ratio can predict cardiac events and all-cause mortality in patients undergoing hemodialysis. Ann. Nutr. Metab..

[51] Low S., Ng T.P., Goh K.S., Moh A., Khoo J., Ang K. (2024). Reduced skeletal muscle mass to visceral fat area ratio is independently associated with reduced cognitive function in type 2 diabetes mellitus. J. Diabetes Complications.

[52] Shida T., Akiyama K., Oh S., Sawai A., Isobe T., Okamoto Y. (2018). Skeletal muscle mass to visceral fat area ratio is an important determinant affecting hepatic conditions of non-alcoholic fatty liver disease. J. Gastroenterol.

[53] Park J., Kim S. (2016). Validity of muscle-to-fat ratio as a predictor of adult metabolic syndrome. J. Phys. Ther. Sci..

[54] Liu D., Zhong J., Ruan Y., Zhang Z., Sun J., Chen H. (2021). The association between fat-to-muscle ratio and metabolic disorders in type 2 diabetes. Diabetol. Metab. Syndr..

[55] Grijalva-Eternod C.S., Lawlor D.A., Wells J.C.K. (2013). Testing a capacity-load model for hypertension: disentangling early and late growth effects on childhood blood pressure in a prospective birth cohort. PLOS ONE.

[56] Seo Y., Song H.J., Song Y.R. (2020). Fat-to-muscle ratio as a predictor of insulin resistance and metabolic syndrome in Korean adults. J. Cachexia Sarcopenia Muscle.

[57] Booranasuksakul U., Macdonald I.A., Stephan B.C.M., Siervo M. (2024). Body composition, sarcopenic obesity, and cognitive function in older adults: findings from the National Health and Nutrition Examination Survey (NHANES) 1999–2002 and 2011–2014. J. Am. Nutr. Assoc..

[58] Montagnese C., Nutile T., Marphatia A.A., Grijalva-Eternod C.S., Siervo M., Ciullo M. (2014). Body composition, leg length and blood pressure in a rural Italian population: a test of the capacity-load model. Nutr. Metab. Cardiovasc. Dis..

[59] Kim T.N., Park M.S., Lim K.I., Yang S.J., Yoo H.J., Kang H.J. (2011). Skeletal muscle mass to visceral fat area ratio is associated with metabolic syndrome and arterial stiffness: the Korean Sarcopenic Obesity Study (KSOS). Diabetes Res. Clin. Pract..

[60] Lim K.I., Yang S.J., Kim T.N., Yoo H.J., Kang H.J., Song W. (2010). The association between the ratio of visceral fat to thigh muscle area and metabolic syndrome: the Korean Sarcopenic Obesity Study (KSOS). Clin. Endocrinol. (Oxf)..

[61] Xiao J., Cain A., Purcell S.A., Ormsbee M.J., Contreras R.J., Kim J.-S. (2018). Sarcopenic obesity and health outcomes in patients seeking weight loss treatment. Clin. Nutr. ESPEN.

[62] Gätjens I., Schmidt S.C.E., Plachta-Danielzik S., Bosy-Westphal A., Müller M.J. (2021). Body composition characteristics of a load-capacity model: age-dependent and sex-specific percentiles in 5- to 17-year-old children. Obes. Facts..

[63] Wang N., Sun Y., Zhang H., Chen C., Wang Y., Zhang J. (2021). Total and regional fat-to-muscle mass ratio measured by bioelectrical impedance and risk of incident type 2 diabetes. J. Cachexia Sarcopenia Muscle.

[64] Auyeung T.W., Lee J.S.W., Leung J., Kwok T., Woo J. (2013). Adiposity to muscle ratio predicts incident physical limitation in a cohort of 3,153 older adults—an alternative measurement of sarcopenia and sarcopenic obesity. Age (Dordr).

[65] Yu B., Sun Y., Du X., Zhang H., Chen C., Tan X. (2023). Age-specific and sex-specific associations of visceral adipose tissue mass and fat-to-muscle mass ratio with risk of mortality. J. Cachexia Sarcopenia Muscle.

[66] Chen Y.-Y., Fang W.-H., Wang C.-C., Kao T.-W., Yang H.-F., Wu C.-J. (2019). Fat-to-muscle ratio is a useful index for cardiometabolic risks: a population-based observational study. PLOS ONE.

[67] Zhou R., Chen H.W., Lin Y., Li F.R., Zhong Q., Huang Y.N. (2023). Total and regional fat/muscle mass ratio and risks of incident cardiovascular disease and mortality. J. Am. Heart. Assoc..

[68] Wang W., Luo Y., Zhuang Z., Song Z., Huang N., Li Y. (2022). Total and regional fat-to-muscle mass ratio and risks of incident all-cause dementia, Alzheimer’s disease, and vascular dementia. J. Cachexia Sarcopenia Muscle.

[69] Ramírez-Vélez R., Garcia-Hermoso A., Prieto-Benavides D.H., Correa-Bautista J.E., Quino-Ávila A.C., Rubio-Barreto C.M. (2019). Muscle mass to visceral fat ratio is an important predictor of the metabolic syndrome in college students. Br. J. Nutr..

[70] Li L., Zhong H., Shao Y., Zhou X., Hua Y., Chen M. (2023). Association between lean body mass to visceral fat mass ratio and bone mineral density in United States population: a cross-sectional study. Arch. Public Health.

[71] Johnson Stoklossa C.A., Sharma A.M., Forhan M., Siervo M., Padwal R.S., Prado C.M. (2017). Prevalence of sarcopenic obesity in adults with class II/III obesity using different diagnostic criteria. J. Nutr. Metab.

[72] Luo Y., Wang Y., Tang S., Xu L., Zhao X., Han M. (2024). Prevalence of sarcopenic obesity in the older non-hospitalized population: a systematic review and meta-analysis. BMC Geriatr.

[73] Liu C., Wong P.Y., Chung Y.L., Chow S.K., Cheung W.H., Law S.W. (2023). Deciphering the “obesity paradox” in the elderly: a systematic review and meta-analysis of sarcopenic obesity. Obes. Rev..

[74] Gao Q., Mei F., Shang Y., Hu K., Chen F., Zhao L. (2021). Global prevalence of sarcopenic obesity in older adults: a systematic review and meta-analysis. Clin. Nutr..

[75] Donini L.M., Busetto L., Bischoff S.C., Cederholm T., Ballesteros-Pomar M.D., Batsis J.A. (2022). Definition and diagnostic criteria for sarcopenic obesity: ESPEN and EASO consensus statement. Obes. Facts..

[76] Donini L.M., Busetto L., Bischoff S.C., Cederholm T., Ballesteros-Pomar M.D., Batsis J.A. (2022). Definition and diagnostic criteria for sarcopenic obesity: ESPEN and EASO consensus statement. Clin. Nutr..

[77] Gortan Cappellari G., Guillet C., Poggiogalle E., Ballesteros Pomar M.D., Batsis J.A., Boirie Y. (2023). Sarcopenic obesity research perspectives outlined by the Sarcopenic Obesity Global Leadership Initiative (SOGLI) – proceedings from the SOGLI consortium meeting in Rome November 2022. Clin. Nutr..

[78] Booranasuksakul U., Tsintzas K., Macdonald I., Stephan B.C.M., Siervo M. (2024). Application of a new definition of sarcopenic obesity in middle-aged and older adults and association with cognitive function: findings from the National Health and Nutrition Examination Survey 1999–2002. Clin. Nutr. ESPEN.

[79] Scott D., Blyth F., Naganathan V., Le Couteur D.G., Handelsman D.J., Waite L.M. (2023). Sarcopenia prevalence and functional outcomes in older men with obesity: comparing the use of the EWGSOP2 sarcopenia versus ESPEN-EASO sarcopenic obesity consensus definitions. Clin. Nutr..

[80] Li R., Chen X., Tang H., Luo S., Lian R., Zhang W. (2024). Sarcopenic obesity and falls in older adults: a validation study of ESPEN/EASO criteria and modifications in Western China communities, Arch. Gerontol. Geriatr.

[81] Mikhail N. (2009). The metabolic syndrome: insulin resistance. Curr. Hypertens. Rep. Inc..

[82] Egan B.M., Greene E.L., Goodfriend T.L. (2001). Insulin resistance and cardiovascular disease. Am J Hypertens.

[83] Ormazabal V., Nair S., Elfeky O., Aguayo C., Salomon C., Zuñiga F.A. (2018). Association between insulin resistance and the development of cardiovascular disease. Cardiovasc. Diabetol..

[84] Srikanthan P., Hevener A.L., Karlamangla A.S. (2010). Sarcopenia exacerbates obesity-associated insulin resistance and dysglycemia: findings from the National Health and Nutrition Examination Survey III. PLOS ONE.

[85] Lee C.G., Boyko E.J., Strotmeyer E.S., Lewis C.E., Cawthon P.M., Hoffman A.R. (2011). Association between insulin resistance and lean mass loss and fat mass gain in older men without diabetes mellitus. J. Am. Geriatr. Soc..

[86] Kim K., Park S.M. (2018). Association of muscle mass and fat mass with insulin resistance and the prevalence of metabolic syndrome in Korean adults: a cross-sectional study. Sci. Rep..

[87] Racette S.B., Evans E.M., Weiss E.P., Hagberg J.M., Holloszy J.O. (2006). Abdominal adiposity is a stronger predictor of insulin resistance than fitness among 50–95 year olds. Diabetes Care.

[88] Wilson M.-M.G., Morley J.E. (2003). Invited review: aging and energy balance. J. Appl. Physiol..

[89] Yaribeygi H., Maleki M., Sathyapalan T., Jamialahmadi T., Sahebkar A. (2021). Pathophysiology of physical inactivity-dependent insulin resistance: a theoretical mechanistic review emphasizing clinical evidence. J. Diabetes Res..

[90] Nunes E.A., Colenso-Semple L., McKellar S.R., Yau T., Ali M.U., Fitzpatrick-Lewis D. (2022). Systematic review and meta-analysis of protein intake to support muscle mass and function in healthy adults. J. Cachexia Sarcopenia Muscle.

[91] Lancet The (2006). Curbing the obesity epidemic. Lancet..

[92] Morton R.W., Traylor D.A., Weijs P.J.M., Phillips S.M. (2018). Defining anabolic resistance: implications for delivery of clinical care nutrition. Curr. Opin. Crit. Care..

[93] Hong S., Choi K.M. (2020). Sarcopenic obesity, insulin resistance, and their implications in cardiovascular and metabolic consequences. Int. J. Mol. Sci..

[94] Müller M.J., Lagerpusch M., Enderle J., Schautz B., Heller M., Bosy-Westphal A. (2012). Beyond the body mass index: tracking body composition in the pathogenesis of obesity and the metabolic syndrome. Obes. Rev..

[95] Batsis J.A., Villareal D.T. (2018). Sarcopenic obesity in older adults: aetiology, epidemiology and treatment strategies. Nat. Rev. Endocrinol..

[96] Gerdts E., Regitz-Zagrosek V. (2019). Sex differences in cardiometabolic disorders. Nat. Med..

[97] Rodgers J.L., Jones J., Bolleddu S.I., Vanthenapalli S., Rodgers L.E., Shah K. (2019). Cardiovascular risks associated with gender and aging. J. Cardiovasc. Dev. Dis..

