# Supplementary material for: Beyond Traditional Body Composition Metrics: Load-Capacity Indices Emerge as Predictors of Cardiometabolic Outcomes—A Systematic Review and Meta-Analysis
Source: Adv Nutr. 2025 Jan 3;16(2):100364. doi: 10.1016/j.advnut.2024.100364 (PMC11808523; doi:10.1016/j.advnut.2024.100364)
Supplement: multimedia component 1 [file mmc1.pdf]

Beyond Traditional Body Composition Metrics: Load-Capacity Indices Emerge as Predictors of Cardiometabolic Outcomes – A Systematic Review and Meta-Analysis

Zhongyang Guan

CONTENTS

Supplementary Table 1. Example search strategy in databases ········ 2

Supplementary Figure 1. Risk of bias assessment of the included studies ········ 3

Supplementary Figure 2. Funnel plot for publication bias ········ 4

Supplementary Figure 3. Forest plots for the meta-analyses (RVE) of (A) SO prevalence and (B) the association between LCIs and cardiometabolic outcomes ········ 5

Supplementary Table 4. Sensitive analysis (leave-one-out) for the meta-analysis of (A) SO prevalence and (B) the association between LCIs and cardiometabolic outcomes ········ 7

Supplementary Table 2. Characteristics of the included studies: See additional Excel document.

Supplementary Table 3. Abbreviations list of Supplementary Table 2: See additional Excel document.

Supplementary Table 1 Example search strategy in databases.

Database	Search strategy
Pubmed	(“capacity load model”[All Fields] OR “capacity load model”[MeSHTerms] OR (“load capacity model”[All Fields] OR “load capacity model”[MeSHTerms] OR “ratio”[All Fields] OR “ratio [MeSH Terms] OR (“sarcopeni*”[MeSH Terms] OR “sarcopeni*”[All Fields] OR “obes*”[MeSH Terms] OR “obes*”[All Fields] OR “sarcopen* obes*”[MeSH Terms] OR “sarcopen* obes*”[All Fields] OR “low muscle mass”[MeSH Terms] OR “low muscle mass”[All Fields] OR "adipos*"[MeSH Terms] OR "adipos*"[All Fields] OR "fat*"[MeSH Terms] OR "fat*"[All Fields]

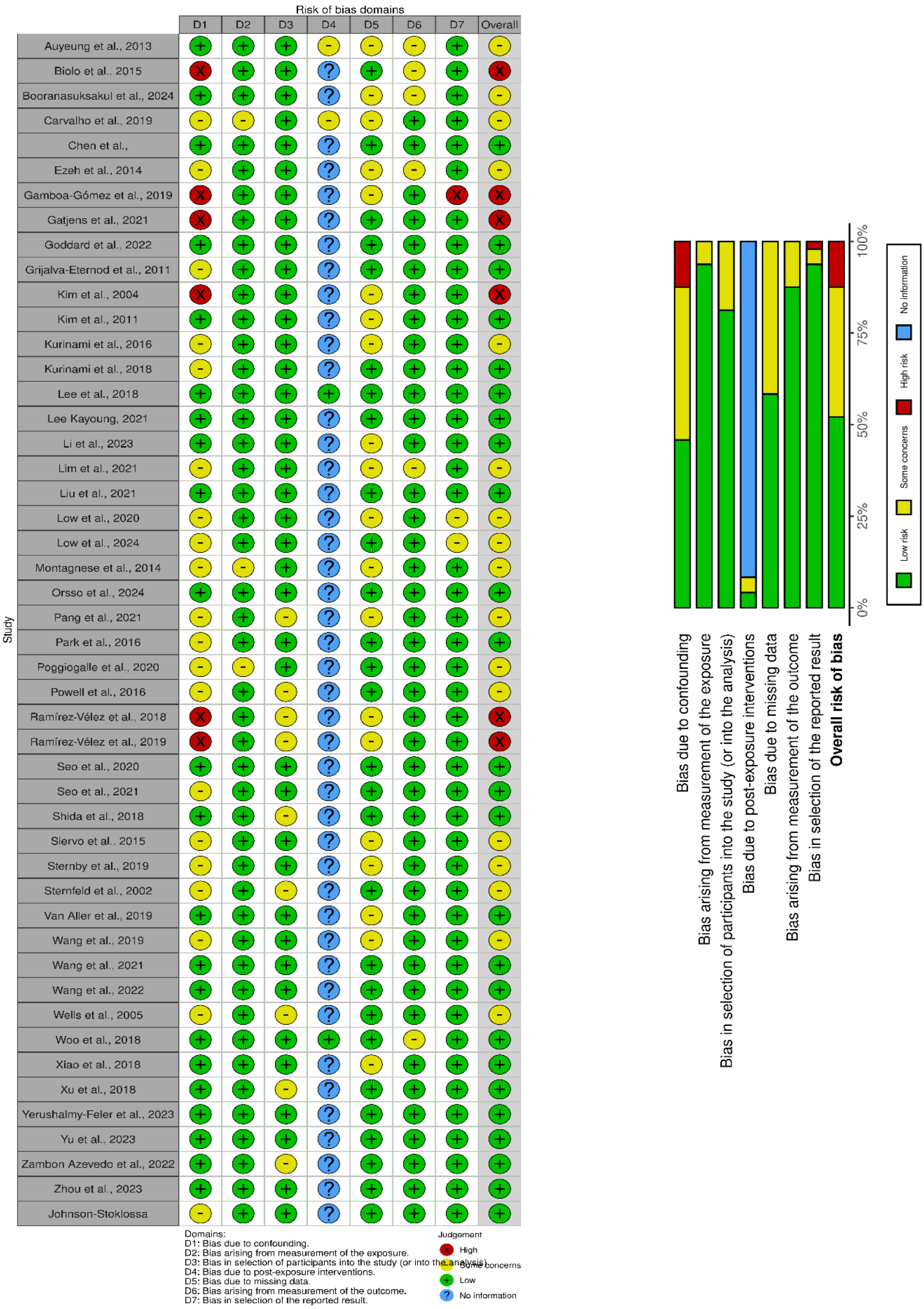

Supplementary Figure 1 Risk of bias assessment of the included studies

A B C

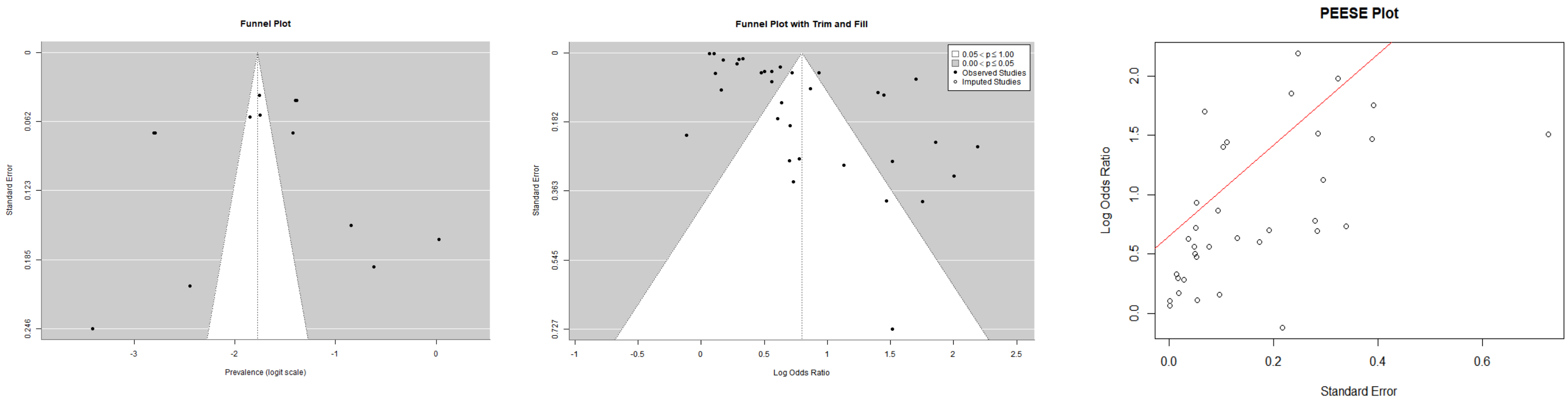

Egger's test: p=0.715; Begg's test: p=0.2721

Egger's test: p<0.0001; Begg's test: p=0.0464

OR (corrected): 1.92 (95% CI: 1.56 to 2.37)

Supplementary Figure 2 Funnel plot for publication bias: (A) Meta-analysis of SO prevalence. (B) Meta-analysis of the association between LCIs and cardiometabolic outcomes. (C) PEESE plot.

A

Author and year	LCI	Sample size	SO Prevalence (95%CI)
Van Aller et al., 2019	TrFM/ASM	3577	5.8 (5.1 to 6.6)
Van Aller et al., 2019	FM/FFM	3577	5.7 (5.0 to 6.5)
Low et al., 2020	FM/FFM	1235	19.4 (17.3 to 21.7)
Goddard et al., 2022	TrFM/ASM	5397	14.7 (13.8 to 15.7)
Pang et al., 2021	FM/FFM	535	3.2 (2.0 to 5.0)
Powell et al., 2016	FM/FFM	3441	19.8 (18.5 to 21.2)
Powell et al., 2016	VAT/FFMI	3441	20.0 (18.7 to 21.4)
Poggiogalle et al., 2020	TrFM/ASM	314	8.0 (5.5 to 11.5)
Poggiogalle et al., 2020	FM/FFM	314	8.0 (5.5 to 11.5)
Biolo et al., 2015	FM/FFM	200	30.0 (24.1 to 36.7)
Booranasuksakul et al., 2024	TrFM/ASM	2544	14.8 (13.5 to 16.3)
Booranasuksakul et al., 2024	FM/FFM	2544	13.6 (12.3 to 15.0)
Xiao et al., 2018	FM/FFM	144	50.7 (42.6 to 58.7)
Johnson-Stoklossa et al., 2017	FM/FFM	120	35.0 (26.9 to 44.0)
**Subgroup**	TrFM/ASM	11 832	10.2 (6.3 to 16.0)
**Subgroup**	FM/FFM	12 110	16.3 (8.7 to 28.4)
**Subgroup**	VAT/FFMI	3441	20.0 (18.7 to 21.4)
**Overall (RVE)**	NA	27 383	16.3 (9.5 to 26.5)

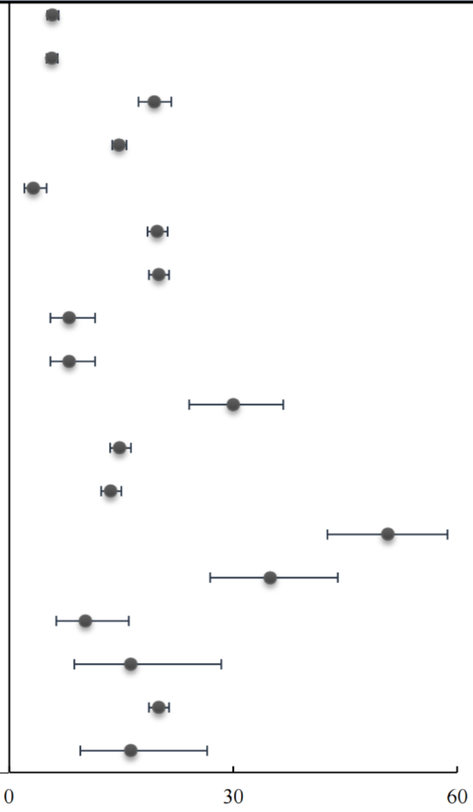

B

Author and year	LCI	Cardiometabolic outcome	OR (95%CI)
Orsso et al., 2024	FM/FFM	Dyslipidaemia	4.54 (1.09 to18.82)
Xu et al., 2018	FM/MM	MetS	8.94 (5.53 to 14.59)
Park et al., 2016	FM/MM	MetS	1.75 (1.46 to 1.97)
Park et al., 2016	FM/MM	Diabetes	1.07 (1.06 to 1.07)
Liu et al., 2021	FM/MM	MetS	4.35 (2.03 to 9.34)
Powell et al., 2016	FM/FFM	MetS	4.25 (3.42 to 5.27)
Powell et al., 2016	VAT/FFMI	MetS	4.06 (3.31 to 4.97)
Kurinami et al., 2016	FM/MM	Insulin resistance	1.12 (1.01 to 1.25)
Gamboa-Gómez et al., 2019	FM/LM	Diabetes	6.4 (5.7 to 14.3)
Seo et al., 2021	VFA/PMA	Diabetes	2.02 (1.39 to 2.94)
Seo et al., 2021	VFA/PMA	Hypertension	2.01 (1.21 to 3.68)
Seo et al., 2021	VFA/TMA	Diabetes	1.83 (1.3 to 2.57)
Seo et al., 2021	VFA/TMA	Hypertension	2.18 (1.26 to 3.77)
Woo et al., 2018	FM/ASM	CVDs	1.17 (0.97 to 1.42)
Woo et al., 2018	FM/ASM	Diabetes	0.89 (0.58 to 1.36)
Wang et al., 2019	VFA/SM	Diabetes	3.09 (1.73 to 5.52)
Wang et al., 2019	VFA/SM	MetS	7.4 (3.92 to 13.97)
Seo et al., 2020	FM/LM	MetS	5.48 (4.79 to 6.27)
Seo et al., 2020	FM/LM	Insulin resistance	2.54 (2.29 to 2.81)
Seo et al., 2020	FM/LM	Dyslipidaemia	2.06 (1.86 to 2.28)
Seo et al., 2020	FM/LM	High cholesterol	1.65 (1.5 to 1.82)
Kim et al., 2011	VFA/ASM	MetS	4.55 (2.6 to 7.96)
Lim et al., 2010	VFA/TMA	MetS	5.77 (2.68 to 12.42)
Wang et al., 2021	FM/MM	Diabetes	1.35 (1.31 to 1.4)
Wang et al., 2021	TrFM/TrMM	Diabetes	1.11 (1.11 to 1.12)
Wang et al., 2021	LFM/LMM	Diabetes	1.39 (1.35 to 1.43)
Wang et al., 2021	AFM/AMM	Diabetes	1.19 (1.14 to 1.23)
Xiao et al., 2018	FM/FFM	High cholesterol	2.08 (1.07 to 4.04)
Chen et al., 2019	FM/MM	MetS	2.38 (1.98 to 2.86)
Chen et al., 2019	FM/MM	Diabetes	1.89 (1.46 to 2.44)
Zhou et al., 2023	FM/MM	CVDs	1.75 (1.59 to 1.92)
Zhou et al., 2023	TrFM/TrMM	CVDs	1.33 (1.26 to 1.41)
Zhou et al., 2023	AFM/AMM	CVDs	1.87 (1.74 to 2.01)
Zhou et al., 2023	LFM/LMM	CVDs	1.61 (1.45 to 1.78)
**Subgroup**	NA	MetS	4.24 (3.07 to 5.86)
**Subgroup**	NA	Diabetes	1.64 (1.2 to 2.23)
**Subgroup**	NA	Other outcomes	1.7 (1.46 to 1.98)
**Subgroup**	FM/MM	NA	2.03 (1.33 to 3.08)
**Subgroup**	FM/FFM	NA	3.41 (2.01 to 5.81)
**Subgroup**	Other LCIs	NA	2.22 (1.73 to 2.85)
**Overall (RVE)**	NA	NA	2.56 (1.79 to 3.65)

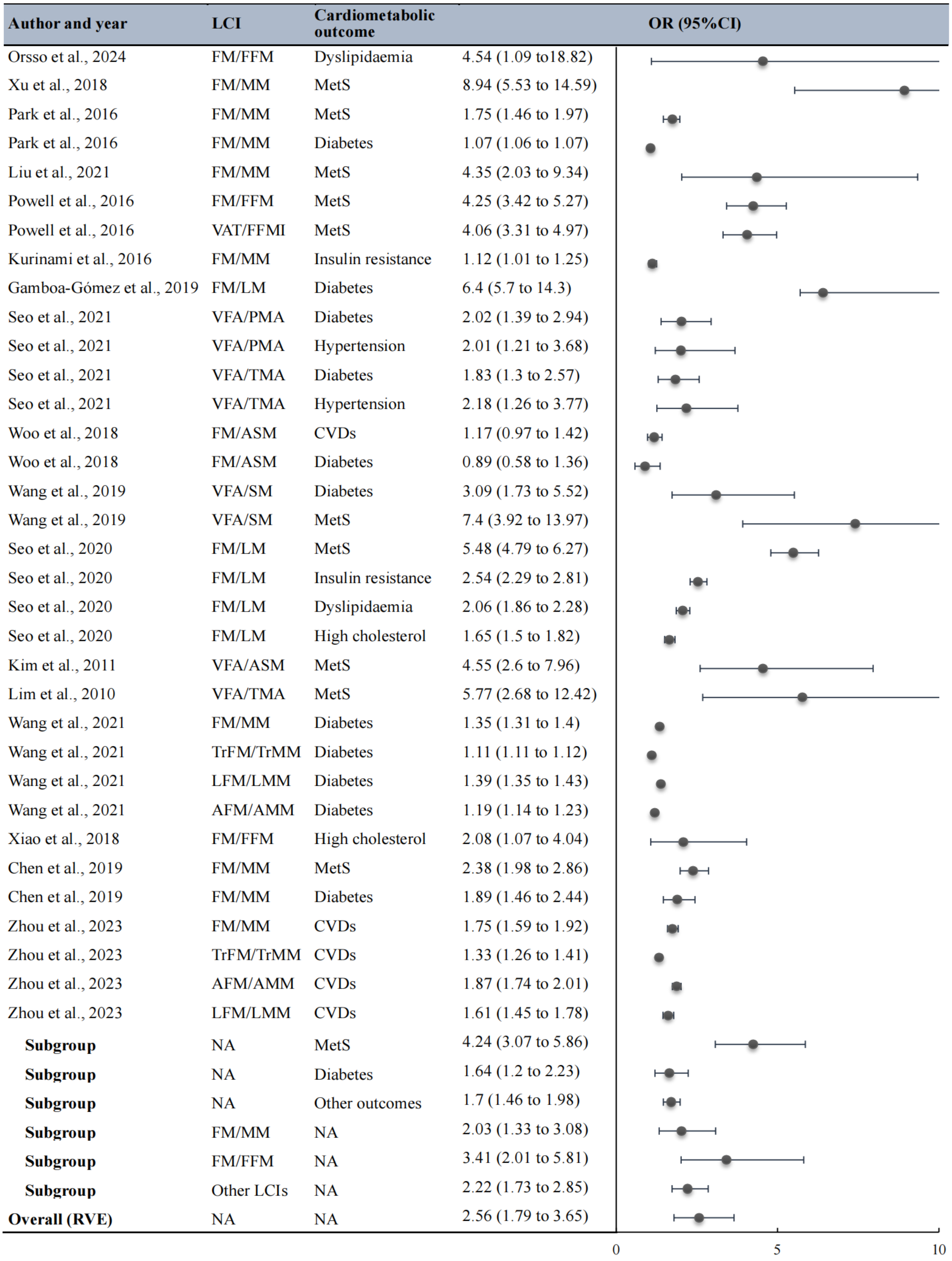

Supplementary Figure 3. Forest plots for the meta-analyses (RVE) of (A) SO prevalence and (B) the association between LCIs and cardiometabolic outcomes.

Supplementary Table 4. Sensitive analysis (leave-one-out) for the meta-analysis of (A) SO prevalence and (B) the association between LCIs and cardiometabolic outcomes.

A

Omitted	Prevalence	CI Lower	CI Upper	I^2 (%)	Q
Study 1A	15.50%	10.03%	23.18%	99.42	600.34
Study 1B	15.51%	10.05%	23.19%	99.42	596.95
Study 2	14.14%	8.89%	21.75%	99.47	818.8
Study 3	14.47%	9.08%	22.26%	99.38	836.21
Study 4	16.03%	10.78%	23.17%	99.36	789.86
Study 5A	14.12%	8.88%	21.72%	99.40	768.45
Study 5B	14.11%	8.88%	21.70%	99.40	762.2
Study 6A	15.12%	9.63%	22.94%	99.49	825.17
Study 6B	15.12%	9.63%	22.94%	99.49	825.17
Study 7	13.63%	8.71%	20.70%	99.47	804.78
Study 8A	14.46%	9.07%	22.25%	99.45	837.12
Study 8B	14.55%	9.14%	22.38%	99.46	832.07
Study 9	12.89%	8.73%	18.62%	99.28	725.86
Study 10	13.45%	8.68%	20.25%	99.44	803.85

B

Omitted	OR	CI Lower	CI Upper	I^2 (%)	Q
Study 1	2.20	1.79	2.70	99.95	2666.31
Study 2	2.12	1.75	2.56	99.94	2598.70
Study 3A	2.24	1.82	2.76	99.95	2633.82
Study 3B	2.27	1.85	2.78	99.67	2346.93
Study 4	2.18	1.78	2.68	99.95	2657.70
Study 5A	2.17	1.77	2.66	99.95	2520.82
Study 5B	2.17	1.77	2.67	99.95	2512.44
Study 6	2.27	1.85	2.78	99.95	2670.03
Study 7	2.15	1.76	2.62	99.95	2616.04
Study 8A	2.23	1.81	2.74	99.95	2660.12
Study 8B	2.23	1.81	2.74	99.95	2665.64
Study 8C	2.23	1.81	2.75	99.95	2661.66
Study 8D	2.22	1.80	2.74	99.95	2664.18
Study 9A	2.26	1.84	2.78	99.95	2669.75
Study 9B	2.28	1.86	2.78	99.95	2669.11
Study 10A	2.20	1.79	2.71	99.95	2658.02
Study 10B	2.15	1.76	2.61	99.95	2635.67
Study 11A	2.15	1.76	2.62	99.95	2125.99
Study 11B	2.21	1.79	2.72	99.95	2415.36
Study 11C	2.23	1.81	2.75	99.95	2525.93
Study 11D	2.24	1.82	2.76	99.95	2603.82
Study 12	2.17	1.77	2.67	99.95	2645.50
Study 13	2.17	1.77	2.65	99.95	2652.24
Study 14A	2.26	1.83	2.78	99.95	2528.59
Study 14B	2.27	1.85	2.78	99.67	2660.64
Study 14C	2.25	1.83	2.77	99.95	2422.00
Study 14D	2.26	1.84	2.78	99.95	2655.20
Study 15	2.22	1.81	2.74	99.95	2666.61
Study 16A	2.22	1.80	2.73	99.95	2603.10
Study 16B	2.23	1.81	2.75	99.95	2653.28
Study 17A	2.24	1.82	2.76	99.95	2578.48
Study 17B	2.26	1.84	2.78	99.95	2628.04
Study 17C	2.23	1.81	2.75	99.95	2464.95
Study 17D	2.24	1.82	2.76	99.95	2618.15
